# FastTree 2 – Approximately Maximum-Likelihood Trees for Large Alignments

**DOI:** 10.1371/journal.pone.0009490

**Published:** 2010-03-10

**Authors:** Morgan N. Price, Paramvir S. Dehal, Adam P. Arkin

**Affiliations:** 1 Physical Biosciences Division, Lawrence Berkeley National Lab, Berkeley, California, United States of America; 2 Virtual Institute of Microbial Stress and Survival, Lawrence Berkeley National Lab, Berkeley, California, United States of America; 3 Department of Bioengineering, University of California, Berkeley, California, United States of America; Providence Health Care, Canada

## Abstract

**Background:**

We recently described FastTree, a tool for inferring phylogenies for alignments with up to hundreds of thousands of sequences. Here, we describe improvements to FastTree that improve its accuracy without sacrificing scalability.

**Methodology/Principal Findings:**

Where FastTree 1 used nearest-neighbor interchanges (NNIs) and the minimum-evolution criterion to improve the tree, FastTree 2 adds minimum-evolution subtree-pruning-regrafting (SPRs) and maximum-likelihood NNIs. FastTree 2 uses heuristics to restrict the search for better trees and estimates a rate of evolution for each site (the “CAT” approximation). Nevertheless, for both simulated and genuine alignments, FastTree 2 is slightly more accurate than a standard implementation of maximum-likelihood NNIs (PhyML 3 with default settings). Although FastTree 2 is not quite as accurate as methods that use maximum-likelihood SPRs, most of the splits that disagree are poorly supported, and for large alignments, FastTree 2 is 100–1,000 times faster. FastTree 2 inferred a topology and likelihood-based local support values for 237,882 distinct 16S ribosomal RNAs on a desktop computer in 22 hours and 5.8 gigabytes of memory.

**Conclusions/Significance:**

FastTree 2 allows the inference of maximum-likelihood phylogenies for huge alignments. FastTree 2 is freely available at http://www.microbesonline.org/fasttree.

## Introduction

Inferring evolutionary relationships, or phylogenies, from families of related DNA or protein sequences is a central method in computational biology. Sequence-based phylogenies are widely used to understand the evolutionary relationships of organisms and to analyze the functions of genes.

The largest gene families already contain tens to hundreds of thousands of representatives, and with the rapid improvements in DNA sequencing, we expect even larger data sets to arrive soon. Large families can be aligned with profile-based methods that scale linearly with the number of sequences (http://hmmer.janelia.org/; [1]). However, most methods for inferring phylogenies from these alignments scale as O(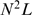) or worse, where 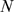 is the number of sequences and 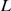 is the length (width) of the alignment. Thus, inferring phylogenies has become computationally challenging.

We recently described a scalable method for inferring phylogenies, FastTree 1.0 [2]. FastTree 1.0 is based on the “minimum-evolution” principle – it tries to find a topology that minimizes the amount of evolution, or the sum of the branch lengths. FastTree 1.0 uses a heuristic variant of neighbor joining [3], [4] to quickly find a starting tree and uses nearest-neighbor interchanges (NNIs) to refine the topology. (A nearest-neighbor interchange swaps a node and its neighbor; for example, it might change ((A,B),C,D) to ((A,C),B,D) or ((A,D),B,C).) FastTree implements these operations in O(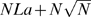) space, where 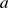 is the number of characters in the alphabet, by storing profiles of subtrees instead of distances between them. This requires far less memory than storing the pairwise distances, which is necessary for neighbor joining and related approaches. This also allows for heuristics that reduce the theoretical running time to O(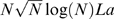). (FastTree 1.0 also included some O(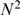) steps, but these have since been removed, see http://www.microbesonline.org/fasttree/ChangeLog.) In comparison, computing all pairwise distances, which is required with most minimum-evolution approaches, requires O(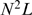) time. The main limitation of FastTree 1.0, as compared to other minimum-evolution methods, is that it does not correct distances for multiple substitutions during its initial neighbor joining phase. However, this is more than made up for by the NNIs. In practice, FastTree 1.0 is more accurate than most other minimum-evolution methods, but not as accurate as maximum-likelihood methods [2].

In the maximum-likelihood (ML) approach, evolution is explicitly modeled with a transition rate matrix, and the tree that best explains the data – the tree with the highest likelihood – is the best tree [5]. The ML criterion ranks the trees but does not specify how to find a good topology. Because ML phylogenetic inference is NP complete [6], no practical method can guarantee that it will find the optimal topology for a large alignment. The most scalable ML methods, such as PhyML and RAxML, begin with a starting tree produced by a faster method, and try to increase the likelihood by optimizing individual branch lengths and performing local rearrangements [7]–[9]. By re-optimizing only a few branch lengths at each move, the cost of considering or performing a move can be reduced to O(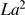) time, where 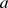 is the size of the alphabet. However, in practice, the number of moves grows as roughly O(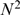), and the optimization steps are inherently slow because they require numerical solving and iteration. This explains why both PhyML and RAxML can take over a day for just 1,000 protein sequences. Estimating the reliability of the tree with the bootstrap [10] generally increases the computational requirements another 100-fold (although this can be reduced by reusing computations across replicates [11]).

Here, we describe FastTree 2, a tool for inferring ML trees for large alignments. Besides constructing an initial tree with neighbor joining and improving it with minimum-evolution NNIs, FastTree 2 uses minimum-evolution subtree-pruning-regrafting (SPRs) [8], [12] and ML NNIs to further improve the tree. (In subtree-pruning-regrafting, a subtree is removed from the tree and reinserted elsewhere, e.g., pruning and regrafting C might change ((A,B),(C,D),E) to ((A,(B,C)),D,E).) FastTree 2 uses heuristics to reduce the search space and hence to maintain the scalability of both stages. Another justification for reducing the search space is that intensive tree search often finds small improvements in the tree's length or likelihood, but these changes may not be statistically or biologically significant (e.g., [13]). Briefly, FastTree's key heuristics are:

- It uses “linear SPRs” to consider just 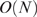 of the 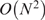 possible SPR moves. At each node, it examines the shortest SPRs first and then extends the most promising candidates.
- It searches for SPR moves for every subtree just twice, instead of iterating until convergence.
- During the ML phase, it limits the ML NNIs to at most 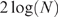 rounds; in practice, it converges before it reaches this limit, but the limit ensures a predictable running time.
- It limits the effort to optimize model parameters and branch lengths.
- It abandons optimization for NNI moves that seem, after partial optimization, to significantly lower the likelihood.
- It does not try to improve parts of the tree that did not improve in recent rounds.

To account for the variation in rates across sites, FastTree uses the “CAT” approximation [14] rather than the standard discrete gamma model with four rates (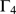) [15]. Some sites evolve much more slowly than others, and the ideal way to account for this is to integrate the likelihood at each site over the (unknown) relative evolutionary rate of that site, using a prior distribution over the relative rates such as a gamma distribution. However, these integrals are analytically intractable and computationally prohibitive. The “
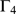
” approach is to use four rate categories to approximate the continuous gamma distribution. However, 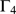 still requires four times more CPU time and memory than a model with no rate variation across sites. Furthermore, for large alignments, the data tightly constrains the rate at each site. Thus, it is much faster, and just as accurate, to use a good estimate of the rate at each site (CAT) rather than to sum over four potential rates (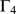) [14]. FastTree selects the most likely rate for each site from among 20 fixed possibilities.

Because of the heuristics, FastTree 2 is not guaranteed to reach a locally optimal likelihood in tree space. However, at each step it does guarantee that the likelihood increases (under the CAT approximation). Thus, FastTree 2 is an approximately-maximum-likelihood method.

We will show that in practice, FastTree 2 is slightly more accurate than a standard implementation of maximum-likelihood NNIs, PhyML 3 with default settings [16], [17]. Specifically, in simulations, FastTree 2 recovers a higher proportion of true splits, and on genuine alignments, FastTree 2's topologies tend to have higher likelihoods. FastTree's minimum-evolution SPR moves give it a better starting tree than PhyML's starting tree, which is obtained with BIONJ (a weighted variant of neighbor joining [18]). This more than makes up for FastTree's heuristics, which reduce the intensity of search for ML NNIs but have little effect on accuracy. We also confirm that using the CAT approximation instead of the 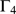 model (which is itself an approximation of the continuous gamma distribution) has little effect on the quality of the tree.

Although FastTree 2 is significantly less accurate than ML methods that use SPR moves, such as PhyML with slower settings or RAxML, most of the splits that disagree are poorly supported, and FastTree is much faster. FastTree 2 can analyze alignments with tens or hundreds of thousands of sequences in under a day on a desktop computer. For alignments with 500 sequences or more, FastTree 2 is at least 100 times faster than either PhyML 3.0 or RAxML 7.2.1. FastTree 2 is faster than RAxML 7 mostly because of less intensive ML search (NNIs instead of SPRs) and because RAxML 7 optimizes branch lengths under the 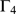 model. However, FastTree also has a faster starting tree, and it initially increases the likelihood more quickly than RAxML 7 does.

Because of its speed, FastTree 2 is suitable for bootstrapping. However, to provide a quicker estimate of the tree's reliability, FastTree 2 provides local support values based on the Shimodaira-Hasegawa (SH) test [16], [17], [19]. FastTree 2 should be useful for reconstructing the tree of life and for analyzing the millions of uncharacterized proteins that are being identified by genome sequencing.

## Results

We compared FastTree's speed and accuracy to those of PhyML 3.0 and RAxML 7, the most popular maximum-likelihood methods. To measure the quality of the resulting trees, we measured the topological accuracy on simulated alignments and the likelihood on genuine biological alignments.

### Topological Accuracy in Simulations

We tested FastTree on simulated protein alignments with 250 to 5,000 sequences [2]. These simulations were derived from diverse gene families that arise in genome-scale studies (“Collections of Orthologous Groups” or COGs, [20]). The simulations include varying evolutionary rates across sites and include realistic placement of gaps. The simulations are available from the FastTree web site (http://microbesonline.org/fasttree/#Sims).

We defined the topological accuracy as the proportion of the splits in the true trees that are recovered by each method. This is the converse of the topological (“Robinson-Foulds”) distance, scaled to range from 0 to 1. As shown in Table 1, FastTree 2 was slightly more accurate than PhyML 3 with default settings (NNI search), and much more accurate than minimum-evolution or parsimony methods, but not as accurate as ML methods that use SPR moves. The differences in accuracy between FastTree 2 and the other methods were statistically significant (all 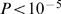, paired 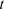 tests).

**Table 1:** Topological accuracy of trees inferred from simulated alignments.

	250	1,250	5,000	78,132
Method	a.a.	a.a.	a.a.	nt.
RAxML 7 (JTT+CAT, SPRs)	90.5%	88.4%	88.4%	–
PhyML 3.0 (JTT+ 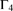 , SPRs)	89.9%	–	–	–
FastTree 2.0.0 (JTT+CAT or JC+CAT)	86.9%	83.7%	84.3%	92.1%
PhyML 3.0 (JTT+ 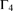 , no SPRs)	86.0%	–	–	–
FastME 2.06 (log-corrected distances, SPRs)	80.5%	78.8%	77.0%	–
FastTree 2.0.0, no ML NNIs	80.4%	78.3%	76.6%	91.4%
BIONJ (ML distances)	77.7%	73.7%	73.1%	–
Parsimony (RAxML)	76.8%	76.5%	69.4%	–
Neighbor joining (log-corrected distances)	76.0%	72.6%	71.6%	66.1%
Clearcut (log-corrected distances)	75.5%	72.3%	71.5%	58.1%

For alignments with 5,000 sequences, we used RAxML 7.2.1 with fast convergence; for smaller alignments we used RAxML 7.0.4. An earlier version of PhyML 3 took up to 4 days for individual simulations with 1,250 sequences, even without 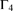, so we did not try to run PhyML 3 with 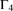 on the larger simulations.

**Figure 1:**
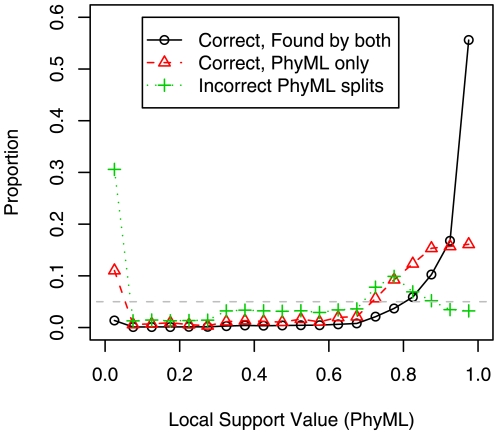
Local support values for splits found by PhyML with SPR moves and/or FastTree. We examined local support values for the splits inferred by PhyML 3.0 with 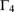 + SPRs on simulated alignments with 250 protein sequences. We classified PhyML's splits as correct and found by both PhyML and FastTree, correct but missed by FastTree, or incorrect. We show the distribution of support values for each class. The right-most bin includes the strongly supported splits (0.95 to 1.0), and the gray dashed line shows the uniform distribution. The support values are PhyML's minimum of the approximate likelihood ratio test [21] and SH-like [16], [17] local supports.

To test the practical significance of the additional true splits that are found by using ML SPR moves, we examined the local support values reported by PhyML 3. We defined “strongly supported” as having both SH-like local supports and approximate likelihood ratio test (aLRT) supports [21] of 95% or higher. Only 16% of the true splits that are found by PhyML 3 with SPR moves but missed by FastTree 2 were strongly supported. The full distribution of support values is shown in Figure 1. Conversely, among the strongly supported splits that were found by PhyML 3 with SPRs but not FastTree, 20% were incorrect. Thus, few of the additional true splits have high support, and of the splits that disagree, even the ones that have high support have a significant probability of being incorrect.

To understand why FastTree 2 was outperforming PhyML 3 with NNI search, we ran PhyML 3 with FastTree's minimum-evolution tree as its starting tree. For the protein simulations with 250 sequences, this improved PhyML's accuracy to 86.8%, which is statistically indistinguishable from FastTree's accuracy of 86.9% (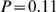, paired 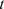 test, 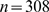). We also confirmed that FastTree's minimum-evolution phase yields more accurate starting trees than either PhyML 3's approach of using BIONJ with maximum-likelihood distances or RAxML's implementation of parsimony (Table 1).

### CAT-Based Branch Lengths and Local Support Values

Because FastTree 2 does not exhaustively optimize the likelihood, and because it reports branch lengths and local support values that were estimated using the CAT approximation, we compared its branch lengths and local support values to 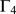-based lengths and supports. Specifically, for the protein simulations with 250 sequences, we re-optimized the branch lengths and computed local SH-like support values for the FastTree topologies with PhyML 3 and 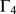. (For both tools, we used the Jones-Taylor-Thorton (JTT) model of amino acid evolution.) PhyML's internal branch lengths were well correlated with those from FastTree (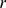 = 0.90). For branch lengths of 1.0 or less, the average difference was just 0.01, and for branch lengths between 0.01 and 1.0, the average percent difference was 13%. For internal branch lengths on correct splits, FastTree agreed slightly better with the true lengths (median absolute difference of 0.062 for PhyML and 0.059 for FastTree). Thus, the CAT approximation gave acceptable branch lengths.

If accurate branch lengths are essential, however, then neither the CAT approximation nor the standard 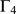 approximation is sufficient. The 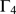 approximation was introduced for alignments with just 10 sequences, and four discrete rate categories may not suffice to give accurate likelihoods on larger alignments [15], [22]. For alignments of 16S ribosomal RNAs, 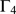 branch lengths can be a factor of two shorter than 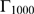 branch lengths (Figure S1). As explained in Figure S1, correcting by the average posterior rate reduces this problem, and FastTree can compute a fast but accurate approximation to 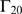-based lengths.

The local SH-like support values also showed a good correlation between FastTree and PhyML (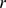 = 0.90). For splits with local support values of at least 0.9 from either FastTree or PhyML, the average absolute difference was just 0.008, which is not much greater than the sampling error. (For example, with 1,000 bootstraps and 95% support, the standard deviation of support values due to sampling is 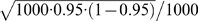 = 0.007.) FastTree was less effective than PhyML in distinguishing correct from incorrect splits, but the difference was slight: the area under the receiver operating curve (AOC) was 0.880 instead of 0.887 (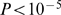, test of [23]).

### Effectiveness of Heuristics

We then examined how the topological accuracy of FastTree 2 is affected by its heuristics. As shown in Table 1, the minimum-evolution phase of FastTree, which uses linear SPRs, is not as accurate as FastME 2, a minimum-evolution method that performs exhaustive SPR moves [8], [12]. FastME computes distances between internal nodes differently from the minimum-evolution phase of FastTree: FastME uses averages of distances between sequences, while FastTree uses distances between profiles, which are averages of sequences. Nevertheless, FastTree 1 with only NNI moves gave very similar results as FastME with only NNI moves [2]. Thus, we attribute the modest difference in accuracy of the minimum-evolution methods with SPRs to FastTree's heuristics. To eliminate this effect, we ran FastTree with the FastME starting tree. To eliminate the effect of FastTree's ML heuristics, we ran it with exhaustive ML NNIs, and with more exhaustive optimization of branch lengths within each NNI (4 rounds of optimizing branch lengths for each quartet, instead of 1–2 rounds). In combination, FastTree 2 with FastME+SPR starting trees and exhaustive NNIs improved the accuracy on simulated alignments with 5,000 protein sequences from 84.3% to 85.0%. This modest effect illustrates that all of FastTree's heuristics have little effect on accuracy, and that removing them would improve the topology little relative to adding ML SPRs (e.g., RAxML 7.2.1 was 88.4% accurate).

We also tested FastTree on simulations with over 78,000 nucleotide sequences. These simulations are derived from a 16S ribosomal RNA alignment (see Methods). The large size of these simulated alignments makes them a stringent test of FastTree's heuristics. In these simulations, FastTree gave much more accurate topologies than exact neighbor joining or Clearcut [24], a faster heuristic variant of neighbor joining (Table 1). (To analyze such large alignments with exact neighbor joining, we used NINJA [25].) To verify that the heuristics in FastTree's neighbor joining phase do not reduce accuracy, we also ran FastTree with the exact neighbor-joining tree as its starting tree, before doing minimum-evolution NNIs and SPRs and ML NNIs. This gave the same accuracy as the regular FastTree or as FastTree with the fastest settings of its heuristics for the neighbor joining phase (-fastest). All three variants found 92.10% of splits correctly.

It may seem surprising that FastTree can reach accurate topologies when it does not compare all pairs of sequences to each other. However, minimum-evolution NNIs and SPRs are “consistent” – they find correct trees, even if the distances contain some errors, as long as the errors are much smaller than the internal branch lengths [26], [27]. In practice, the errors are often larger than the internal branch lengths, but this still probably explains why NNIs and SPRs suffice to find most of the splits correctly.

### Quality of Topologies for Biological Alignments

To confirm that FastTree finds good topologies for genuine alignments, and not just in simulations, we tested it on 16S ribosomal RNAs and on protein families from COG. Although these families are quite large (up to 300,000 or 19,000 members, respectively), we first tested random subsets of just 500 sequences, so that we could run PhyML 3 with 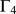. To measure the quality of the topologies from FastTree 2, PhyML 3, and RAxML 7, we re-optimized the branch lengths with a 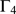 model (using RAxML) and compared the resulting likelihoods. As expected from the simulations, FastTree found better topologies than PhyML 3 with 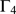 and NNI moves, but not as good as RAxML 7 (Table 2).

**Table 2:** Average log-likelihood for genuine alignments with 500 sequences.

Method	16S	COG
RAxML 7.0.4 (GTR+CAT or JTT+CAT, SPRs)	−168,104	−206,724
FastTree 2.0.0 (GTR+CAT or JTT+CAT)	−168,577	−206,993
PhyML 3.0 (GTR+ 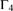 or JTT+ 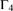 , no SPRs)	−168,603	−207,156

For all topologies, the log likelihood was computed with RAxML 7, re-optimized branch lengths and model parameters, and the GTR+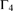 or JTT+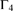 models for 16S or COG, respectively. All differences between FastTree and other methods were statistically significant (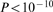) except for the comparison with PhyML on 16S rRNAs (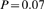, paired 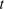 test).

We then tested FastTree and RAxML on larger alignments of 16S rRNAs and COGs. For alignments with thousands of sequences, RAxML 7.0.4 is a bit slow, so we used RAxML 7.2.1, which introduced a fast convergence option as well as other optimizations. With fast convergence, RAxML terminates the search if less than 1% of splits change during a round of SPR moves. As shown in Table 1, for 5,000 proteins, RAxML with fast convergence is nevertheless quite accurate.

On the larger alignments, RAxML 7.2.1's likelihoods were much higher than FastTree's, and all of the differences in likelihood were statistically significant (all 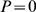, SH test using CONSEL [28]). However, FastTree did find most of the splits in the RAxML topology that had strong support (Table 3). For example, FastTree found 96–98% of RAxML's splits that had global bootstrap of 90% or higher.

**Table 3:** Comparison of RAxML and FastTree's log likelihoods, and the agreement of FastTree with RAxML's well-supported splits, for large genuine alignments.

	16S rRNA	16S rRNA	7 COGs
Number of sequences	4,114	6,718	2,500
RAxML 7's Log Likelihood	−325,581	−481,259	−1,238,666
FastTree 2's Log Likelihood	−328,062	−493,841	−1,240,916
Difference	2,481	12,582	2,251
Well-supported RAxML splits (bootstrap 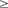 0.9)			
Total in RAxML tree	851	1,124	–
Found by FastTree	837	1,075	–
Weakly-supported RAxML splits (bootstrap 0.8–0.9)			
Total in RAxML tree	265	419	–
Found by FastTree	250	365	–
Locally-supported RAxML splits (SH 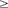 0.95)			
Total in RAxML tree	1,336	1,927	1,018
Found by FastTree	1,033	1,319	889

We ran RAxML with the fast convergence option. All values for COGs are averages over seven families. Log likelihoods for all topologies were computed with RAxML using 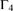 and GTR or JTT. Global bootstrap values are from using the standard bootstrap with RAxML 7.0.4 (from [11]). SH-like local support values for RAxML's topology were computed with FastTree 2, the CAT approximation, and GTR or JTT.

### Running Time and Memory Required

Finally, we compared the computational performance of FastTree, RAxML, and PhyML, on genuine alignments. As shown in Table 4, for alignments with 500 sequences, FastTree is about 100 times faster than RAxML 7.0.4 when using the same model of evolution, and even faster relative to PhyML 3. For alignments with thousands of sequences, FastTree was still 100–800 times faster than RAxML 7.2.1 with fast convergence of SPRs, while PhyML 3 did not complete in a reasonable amount of time.

**Table 4:** Running time and memory usage on genuine alignments.

	Distinct		FastTree 2.0.0			RAxML 7	PhyML 3
Alignment	Sequences	Positions	Model	Hours	GB	Hours	Hours
16S rRNA, subsets	500	1,287 nt.	GTR	0.02	–	2.2	2.9
COGs, subsets	500	65–1,009 a.a.	JTT	0.02	–	5.2	7.2
COGs, subsets	2,500	197–384 a.a.	JTT	0.11	–	61	–
Efflux permeases	8,362	394 a.a.	JTT	0.25	0.35	197	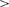 1,200
16S rRNAs, families	15,011	1,287 nt.	GTR	0.66	0.56	64	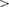 2,000
ABC transporters	39,092	214 a.a.	JTT	1.02	0.96	–	–
16S rRNAs, all	237,882	1,287 nt.	JC	21.8	5.8	–	–

All runs used a single thread of execution. All runs accounted for variable rates across sites, using CAT for RAxML 7 and FastTree 2 or 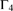 for PhyML 3. All FastTree runs include local SH-like supports and all RAxML runs include branch lengths under 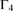. RAxML and PhyML were run without support values (no bootstrap). For random subsets of 500 16S rRNAs or for COGs, we show average running times. For alignments with over 1,000 sequences, we used RAxML 7.2.1's fast convergence option.

For one of the largest alignments existing today, containing 237,882 16S ribosomal RNAs, FastTree took less than a day and 5.8 GB of memory on a desktop computer. For comparison, given that RAxML took over 2 days for just 15,011 sequences, and optimistically assuming O(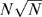) scaling, RAxML would take around half a year for the full 16S alignment. Analyzing such alignments with traditional minimum evolution approaches based on a distance matrix would also be prohibitive – just computing and storing all pairwise distances for these sequences, without computing a topology, would require roughly a day and a half and 113 GB of storage.

All of the FastTree times include the computation of local SH-like support values, while the other tools were run without support values. The local support values do not affect FastTree's running time much. For example, across seven COG alignments with 2,500 protein sequences each, the average time for FastTree to infer a tree is 345 seconds, and the average time for it to compute SH-like supports is 51 seconds. For the full alignment of 237,882 16S rRNAs, the supports required just one hour.

Much of the time in RAxML 7.2.1 is spent optimizing the branch lengths under the 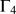 model, even though the CAT approximation is used to search for a good topology. (RAxML can also perform SPR moves under the 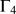 model, but we ran it with SPR moves under the CAT model only, followed by optimizing branch lengths under 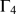, because RAxML 7.2.1 does not report CAT-based branch lengths.) If branch lengths are not required, such as during bootstrapping, then RAxML can be 2–3 times faster than shown in Table 4. For example, for 15,011 16S rRNAs, if the 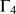 phase is removed, then RAxML 7.2.1 takes 30 hours instead of 64 hours, which is still about 45 times slower than FastTree. The 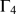 phase of RAxML is also expected to quadruple the memory required. For example, for 15,011 16S rRNAs, FastTree required 0.56 GB of memory, while RAxML with 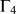 required 2.6 GB.

### Improvement of Likelihood Over Time

**Figure 2:**
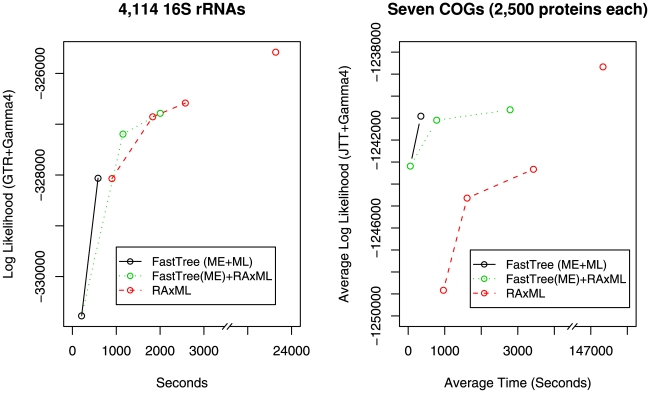
Likelihoods over time for genuine alignments. Each line shows the time it takes a different tool to reach a given likelihood. For the COG alignments, all times and likelihoods are averages over the seven alignments. For FastTree, we show the time and the improvement in likelihood for the minimum-evolution topology and the final (approximately-ML) topology. For RAxML, we show the maximum parsimony starting topology, the first two rounds of SPR moves, and the final topology (note the interrupted 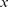 axis). For RAxML with FastTree's (minimum-evolution) starting tree, we show the starting topology and RAxML's first two rounds of SPR moves.

To compare the search strategies of FastTree and RAxML more directly, we compared their improvement in likelihoods over time for a nucleotide alignment of 4,114 16S rRNAs [11] and for seven protein alignments of COG families with 2,500 members. We ran both methods with the CAT approximation and with either the generalized time-reversible (GTR) model of nucleotide substitution or the JTT model of amino acid substitution. We computed likelihoods for intermediate and final trees with RAxML, re-optimized branch lengths, and 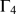. Figure 2 shows the running time and log likelihood for FastTree's minimum-evolution and final tree, for RAxML's initial parsimony tree and successive rounds of SPR moves, and also for RAxML with FastTree's minimum-evolution tree as its starting tree. These times do not include FastTree's support values or RAxML optimizing branch lengths under 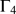.

Given the same starting tree, FastTree's ML phase improved the likelihood by roughly the same amount as one round of RAxML's SPR moves, and in about 40% of the time (Figure 2). FastTree's ML phase also performs about as well as one round of RAxML's SPR moves in finding well-supported splits (Figure S2). We obtained similar results for other large 16S alignments (Table S1). Although this comparison shows that FastTree is initially faster than RAxML, the RAxML's first round of SPR moves is only a fraction of its run time. Most of the difference in speed between FastTree and RAxML is because of RAxML's more thorough search for a better topology and because of RAxML's 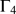 branch lengths.

### Starting Trees: Minimum-Evolution versus Maximum Parsimony

RAxML's parsimony phase was 4–17 times slower than FastTree's minimum evolution phase, and generally slower than FastTree with ML NNIs. FastTree's speed advantage grows with larger alignments (data not shown), which is expected because FastTree should scale as O(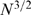) and RAxML's parsimony phase uses randomized stepwise addition, which scales as O(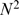), as well as limited parsimony-based SPR moves. There are faster implementations of parsimony, such as RAxML 7.2.5 (which was released after we conducted the above experiments) or TNT [29], but these still scale as O(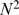). For 15,011 16s RNAs, RAxML 7.2.5's parsimony and FastTree's minimum evolution phase take about the same time (data not shown).

As measured by likelihood, FastTree's minimum-evolution starting trees were much better than RAxML's parsimony starting trees for the COG alignments, but much worse for large 16S rRNA alignments (Figure 2 and Table S1). The differences in likelihood reflects the criterion, and not merely differences in the search strategy: for the COG alignments, the RAxML parsimony starting trees were more parsimonious than FastTree's minimum-evolution trees (average parsimony scores of 281,237 and 283,125, respectively). Conversely, for the 16S alignment with 4,114 sequences, FastTree's minimum-evolution tree was shorter than the parsimony tree (lengths of 43.0 and 44.6, respectively). For this alignment, the minimum-evolution tree's log likelihood was 2,705 worse than parsimony's, yet minimum evolution found more of the strongly-supported splits in the final RAxML tree: minimum evolution found 826 of the 851 splits with a global bootstrap 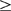
90%, while parsimony found 814 of them. Thus, we are not sure if the difference in likelihood is biologically meaningful.

## Discussion

We have shown that FastTree 2 computes accurate topologies in a reasonable amount of time for alignments with up to hundreds of thousands of sequences. FastTree is open source software and is available at http://microbesonline.org/fasttree. The C source code is extensively documented and contributions are welcome. FastTree trees for every microbial gene family, including families with tens of thousands of members such as ABC transporters, are available at MicrobesOnline (http://microbesonline.org/), along with a “tree-browser” for examining these trees. These trees will be updated from FastTree 1 to FastTree 2 in the next release of MicrobesOnline.

Because DNA sequencing technology is improving rapidly, we expect to have alignments with millions of sequences soon. For these huge alignments, the most computationally demanding step will be the initial neighbor-joining phase. In FastTree 2.0, which is described here, neighbor joining takes O(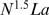) time and O(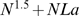) space, while the other stages take at most O(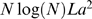) time and O(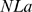) space. For example, for 237,882 16S sequences, the neighbor-joining phase of FastTree 2.0 already takes 10.8 of the 21.8 hours. In FastTree 2.1, we have improved the scaling of time and memory from O(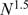) to O(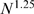), without affecting accuracy in our simulations (data not shown). FastTree 2.1 also supports parallel execution of the key steps in the neighbor-joining phase. To improve scalability further, it might be possible to use a divide-and-conquer method to find clusters of closely related sequences in O(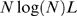) time, as in PartTree [30]. In our simulations, PartTree starting trees do not allow FastTree to reach the same accuracy as FastTree's neighbor-joining starting tree does (data not shown), but a divide-and-conquer approach might still suffice to obtain a partially resolved initial tree.

Such huge families also raise challenges for multiple sequence alignment. We have used profile alignment to avoid the challenges of multiple sequence alignment on large families. This works well for 16S RNAs because Infernal takes advantage of highly conserved secondary structure [1], but we are not sure that it gives accurate results for diverse protein families. In contrast, traditional progressive multiple sequence alignment methods are not scalable because their output grows as O(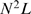): there are O(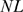) independent insertions, and each insertion requires a new column in the alignment and hence O(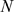) storage. However, Fast Statistical Alignment uses an O(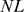) representation, both internally and as an output format [31]. Combining this representation with fast guide tree construction, it should be possible to build progressive multiple sequence alignments with millions of sequences.

Finally, it is not clear how to assess the quality or reliability of such large trees. Different methods gave very different topologies and large differences in likelihood, and yet few of the differences were well-supported by the bootstrap. In fact, a topology with relatively poor likelihood could have relatively good agreement with the best tree. This could indicate that higher-likelihood trees contain many improvements, but that few of the individual improvements are statistically significant. This is expected if there is limited phylogenetic signal. Alternatively, the bootstrap could be too conservative. Local support values do suggest a greater number of significant differences (Table 3), but local support values may be biased upwards because they do not consider all of the alternate topologies. Further study of these questions is needed.

## Materials and Methods

### Minimum-Evolution “Linear” Subtree-Pruning-Regrafting

To reduce the number of SPR moves considered from O(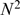) to O(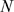), FastTree does just two rounds of “linear SPRs.” For each node, FastTree does an exhaustive search for moves up to length two. It extends each of these moves up to a length of 10 along the best choice at each point along the way.

As suggested by Richard Desper and Olivier Gascuel, FastTree treats each potential SPR move as a sequence of NNIs. The change in tree length for the SPR move is then just the sum of the changes due to the NNIs, much as 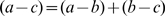.

The change in tree length for an NNI from 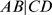 to 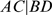, where A, B, C, and D may be subtrees rather than sequences, is estimated by 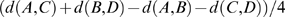. In FastME, which introduced balanced minimum evolution [12], 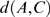 is a topologically weighted average of distances between the members of A and C. In contrast, in FastTree, 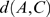 is the log-corrected distance between the profiles for the subtrees A and C, and the profile 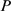 of a subtree is derived from that of its children by 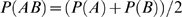. (Although FastTree 1 used weighted joins, as in BIONJ, FastTree 2 uses unweighted joins because they are faster, and the slight effect on accuracy is erased by the ML NNIs.) For nucleotide sequences, the log correction is the Jukes-Cantor correction 
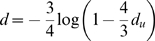
, where 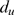 is average dissimilarity of positions across profiles. For amino acid sequences, FastTree uses an empirical log correction similar to that of scoredist [32], 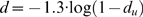, where 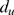 is based on an amino acid dissimilarity matrix derived from the BLOSUM45 similarity matrix.

In FastME, the above formula for the change in tree length is exactly correct because the changes in other branch lengths in the tree can be expressed as combinations of distances that cancel each other out [26]. In FastTree, however, the formula for the change in tree length is an approximation, because the log-corrected distances do not cancel in this way. Nevertheless, FastTree with NNIs and FastME with NNIs give very similar results [2], and computing the exact change in total tree length does not improve the accuracy of FastTree's SPRs (data not shown).

### The Maximum-Likelihood Phase

The key data structures for the maximum likelihood phase are the tree topology, the branch lengths, and the posterior distributions for each internal node. (FastTree stores the tree with a trifurcation at the root, but the placement of the root is not biologically meaningful and does not affect the likelihood [5].) The posterior distribution for an internal node describes the state of the corresponding ancestor, given the branch lengths and the sequences beneath it. For example, for nucleotide data, it stores the probability that a given site was an A, C, G, or T. FastTree stores posterior distributions for 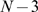 internal nodes (not for the root), and they require O(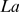) space each, where 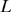 is the alignment's length and 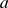 is the number of characters in the alphabet.

The key primitive operations are (1) to compute the joint likelihood of two posterior distributions, given the length between them, and (2) to compute the posterior distribution of a parent node given the posterior distributions of its two children and their two branch lengths. These suffice to compute the likelihood of the tree [5]: for example, the likelihood of the tree (A,B,(C,D)) is 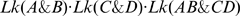 where AB and CD are posterior distributions.

At the beginning of the ML phase, we have a minimum-evolution topology and branch lengths. The steps for the maximum-likelihood phase are:

- Compute an approximate posterior distribution for each node, using the weighted averages of its children. Although the initial posterior distributions are approximate, all future changes to the topology or to the branch lengths will update the posterior distributions to their exact values.
- Optimize all branch lengths for one round, using a simplified model with no parameters (without CAT, and with Jukes-Cantor instead of GTR if GTR was requested).
- Perform one round of ML NNIs, using the simplified model.
- If the GTR model is being used, optimize the nucleotide transition rate parameters, switch from Jukes Cantor to the GTR model and recompute posterior distributions, and optimize all branch lengths for one round with the new model.
- If the CAT model is being used, estimate rate categories for each site, recompute posterior distributions, and optimize all branch lengths for one round with the new model.
- Perform additional rounds of ML NNIs, with subtree skipping and the star topology test.
- Perform a final round of ML NNIs without subtree skipping or the star topology test.
- Optimize all branch lengths for one round.
- Compute SH-like local support values.

#### A round of ML NNIs

**Figure 3:**
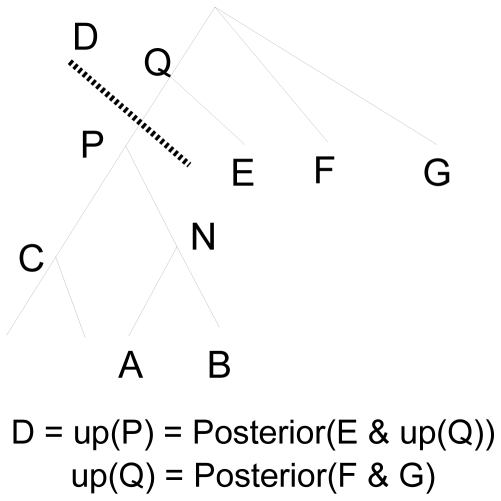
Traversing a tree with up-posteriors. FastTree optimizes the tree near node N by analyzing the posterior distributions for subtrees A, B, and C, as well as the “up-posterior” D.

During each round of NNIs, FastTree visits each node before it visits its parents (depth-first post-order traversal). At each node, it compares the likelihood of the trees 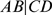, 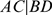, and 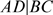, where A and B are its children, C is its sibling, and D is the rest of the tree. During this process, FastTree uses the posterior distributions (or sequences) for A, B, and C and an “up-posterior” D, which represents the rest of the tree. More precisely, the up-posterior D is the posterior distribution of the node's parent N, given all of the nodes that are *not* children of N (see Figure 3). These up-posteriors can be thought of as a way to temporarily reroot the tree at the current location. In particular, the likelihood of the tree can be computed from the posteriors A, B, C, and D.

The up-posterior for a node can be computed from its parent's up-posterior and its sibling's posterior distribution. FastTree only stores these up-posteriors for the path to the root from its current location in the tree, so they take O(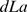) space, where 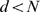 is the maximum depth of the tree. Because FastTree always visits children before their parents, the posterior and up-posterior distributions it uses are up to date, even as the topology changes.

When it visits each node, for each of the three alternate topologies around the node, FastTree optimizes the branch lengths to maximize the likelihood. For the topology 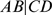, the five initial branch lengths are set from the current tree. For the other topologies, the branch lengths to A, B, C, and D are maintained, as is the internal branch length. Given a quartet (say 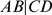), FastTree first optimizes the branch length between AB and CD, and then the branch length leading to A, B, C, and D. FastTree optimizes each branch length to an accuracy of 0.0001 or 0.1%, whichever is greater. These five optimizations define a round of optimization for the quartet. Within a round of optimization, FastTree reuses some of the internal posterior distributions: it needs posterior distributions for AB and CD so that it can optimize the branch length between AB and CD, and then it needs posterior distributions for BCD, ACD, the new posterior distribution for AB given the new branch lengths to A and B, and finally ABD and ABC.

By default, FastTree optimizes the branch lengths within all three quartet topologies for one round. Any topology that is significantly (5 log-likelihood units) worse than the current topology is abandoned after the first round. If more than one topology remains, then the remaining topologies are optimized for another round. After the rounds of optimization are complete, FastTree updates the topology if necessary. In either case, it updates the branch lengths to the re-optimized values and recomputes the posterior distribution for the node.

A difference of 5 in log likelihood may seem like a small difference, so that the heuristic might miss a good change to the topology. However, optimization of branch lengths after the first round usually leads to small improvements in the log likelihood. For example, if we analyze 40 randomly selected 16S rRNAs with FastTree and the GTR+CAT model, and we increase the rounds of branch length optimization to 4 (-mlacc 4), then the average improvement for any NNI is just 1.1 log-likelihood units in the second round of branch length optimization and just 0.04 in rounds 3 and 4 combined. To put these numbers in perspective, differences in log-likelihood of less than 2 are not statistically significant (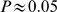, likelihood ratio test), and NNIs with much larger changes in likelihood are common. For the simulated alignments with 5,000 protein sequences, always optimizing for two rounds improved accuracy by a negligible amount (0.03%) and increased the running time by 23%.

#### Optimizing model parameters

After the first round of NNIs, FastTree optimizes any parameters in the model. First, if the GTR model is being used, there are six relative rates to optimize, one for each nucleotide conversion. (The stationary distribution for the transition matrix is set to the empirical frequency of the four nucleotides.) FastTree optimizes the likelihood of the tree (with fixed branch lengths and topology) by numerically optimizing each of the six parameters in the model in turn. With each change in the model, it recomputes all posterior distributions. It then optimizes the six parameters a second time. This does not fully optimize the model parameters, but it gives acceptable results (Table 2).

Second, unless the -nocat option is set, FastTree estimates the rate of evolution at each site. Given the desired number of categories of relative rates 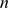, FastTree selects 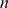 values that are logarithmically spaced between 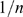 and 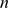. By default, 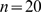, and the relative rates range from 0.05 to 20. For each of these relative rates, FastTree recomputes all posterior distributions and recalculates the log likelihood of the tree at each site. FastTree then uses a Bayesian approach to select which rate to use at each site: FastTree maximizes 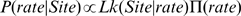, where 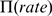 is a gamma-distributed prior. To avoid overfitting, we made the prior more peaked than real rate variation in alignments: the prior has a shape parameter of 3, a scale parameter of 1/3, and a mean of 1. After choosing the rate categories, FastTree scales the rates so that the average rate across all sites is 1.0.

We confirmed that the Bayesian approach to setting the rate categories prevents overfitting on small alignments. For example, on simulated protein alignments with just 10 sequences (from [2]), adding the CAT model improves FastTree's accuracy from 76.2% to 78.0%. (For comparison, PhyML without 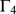 or SPRs was 74.4% accurate [2].) Conversely, on nucleotide simulations with 24 sequences that (unrealistically) do not contain any rate variation across sites (the fast-evolving alignments of [12]), the CAT model only reduces accuracy slightly, from 93.6% to 93.4%. (For comparison, PhyML without 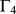 or SPRs was 93.6% accurate [2].)

#### Completing the ML NNIs

In later rounds of NNIs, FastTree uses the more accurate model and it uses two additional heuristics “subtree skipping” and the “star topology test,” which are described below. As discussed in the Results, these heuristics have little effect on accuracy.

If no NNI leads to an improvement of more than 0.1 in the likelihood of any quartet, then FastTree considers the NNIs to have converged. FastTree repeats rounds of NNIs until convergence, up to a limit of 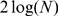 rounds, which takes O(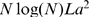) time. This is the slowest part of the ML phase. The limit on rounds ensures a predictable running time, but FastTree usually converges before reaching the limit, even for huge alignments such as 237,882 16S rRNA sequences. We chose a 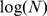 limit so that a misplaced subtree could move all the way across a (roughly balanced) tree, and the factor of 2 is an arbitrary safety factor.

After convergence, FastTree does one final round of ML NNIs with the subtree skipping and the star topology test turned off, as in the first round. We view this as a safety valve for the heuristics. Finally, FastTree does a final round of optimizing the branch lengths and computes the SH-like local supports.

#### Subtree skipping

The intuition behind subtree skipping is that if a subtree has not changed during recent rounds of NNIs, then further attempts to optimize the subtree will be fruitless. Specifically, during ML NNIs, FastTree does not traverse into subtrees that have not seen any significant improvement in likelihood (0.1 log likelihood units) in either of the previous two rounds. Before skipping a subtree, FastTree also checks that none of the nodes adjacent to the parent node were affected by a significantly improving NNI in the previous round. The “subtree skipping” heuristic typically gives a 3-fold speedup, making it the most important of FastTree's ML heuristics. Subtree skipping might be useful for SPR moves as well.

#### Star topology test

If the current topology (A,B,(C,D)) is much better than the star topology (A,B,C,D) then an NNI is unlikely to give an improvement. Specifically, if the current topology is significantly (5 log-likelihood units) more likely than the star topology (after optimizing the internal branch length), then FastTree does not optimize the other branch lengths or consider the two alternate topologies. However, FastTree only uses this heuristic if the node that was unchanged in the last round of NNIs. To approximate the likelihood of the star topology, FastTree uses the likelihood with the minimal internal branch length of 0.0001.

#### Branch lengths

To optimize all branch lengths in the tree at the beginning and end of the ML phase and after optimizing the model parameters, FastTree again uses post-order traversal. At each node, it considers a three-node star topology on the node's children and parent, using the posterior distributions for the two children and the up-posterior for itself. (At the root, it uses all three children instead.) It numerically optimizes these three branch lengths in series for two rounds.

#### SH-like local supports

For each node, the local support is derived from the per-site likelihoods for the current topology and the two alternate (NNI) topologies. For the current topology, FastTree uses the current (already optimized) branch lengths. For the alternate topologies, FastTree optimizes branch lengths for the quartets, as during the NNIs, for up to two rounds. Given the per-site likelihoods for the three topologies, FastTree uses the SH test with 1,000 bootstrap replicates to estimate the confidence in the given split [19]. If there are poorly resolved nodes nearby, then the support values should be interpreted cautiously, because a high-likelihood alternate topology might not have been considered.

#### Low-level optimization of likelihood computations

Whereas RAxML stores likelihood vectors (that is, the joint likelihood of a subtree and of a given character at an internal node), FastTree stores posterior distributions, which are normalized so that each site's values sum to 1. This may improve numerical stability for huge alignments. To reduce memory usage, FastTree stores these vectors in single-precision floating point. Log-likelihoods for the tree or for specific sites are stored with double precision.

Similar to RAxML, FastTree stores the posterior distributions in a rotated form, multiplied by the eigen-matrix of the transition matrix. (For the Jukes Cantor model, this is not necessary.) This reduces the time for likelihood computations from O(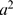) per site to O(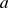), while leaving the cost of computing the posterior distribution at O(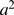) per site (but with a higher constant factor).

While computing the joint likelihood for a pair of posterior distributions, FastTree avoids performing a logarithm at every site by operating on likelihoods instead of log likelihoods. To prevent numerical underflow, FastTree rescales the likelihood by a constant when necessary. It updates a separate (log-likelihood-based) counter whenever it does this. Similarly, when computing the tree's likelihood at each site, for example while optimizing the rate categories, FastTree rescales each site's likelihood if necessary after visiting each node.

FastTree uses SSE2 instructions, a special feature of recent CPUs from Intel and AMD, to operate on 4 single-precision floating point values with one instruction. This speeds up computations for protein alignments by up to 50% (data not shown).

#### Numerical optimization

To find the parameters that optimize the likelihood, FastTree uses Brent's method, a numerical method that iteratively halves the interval it is searching within (http://en.wikipedia.org/wiki/Brents_method). Because Brent's method only operates in one dimension, FastTree optimizes different parameters in turn, and then repeats the rounds of optimization (for example, it optimizes the first branch length, then the second, then the third, then repeats).

FastTree estimates the initial interval to search within from the initial guess 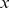 (e.g., the previous length of the branch) and alternate values 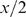 and 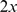. If 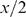 is below the minimum value, it uses the minimum, 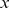, and 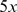 instead. If the initial guess does not bracket the minimum (that is, the middle value is not better than the two endpoints), then FastTree expands the search interval until it does. However, the small interval is usually adequate. FastTree also terminates optimization if the parameter changes by a small amount or by a small proportion. Together, these modifications eliminate about a third of the evaluations of the likelihood.

### Biological and Simulated Alignments

The simulated protein alignments and the genuine COG alignments were described previously [2]. The 16S alignment with 237,882 distinct sequences was taken from GreenGenes [33] (http://greengenes.lbl.gov). The 16S alignment with 15,011 distinct “families” is a non-redundant subset of these sequences (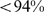 identical). 16S alignments with 500 sequences are also non-redundant random subsets (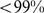 identical). Other large 16S alignments are from [11].

For the 16S-like simulations with 78,132 distinct sequences, we used a maximum-likelihood tree inferred from a non-redundant aligned subset of the full set of 16S sequences (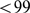
% identity) by an earlier version of FastTree (1.9) with the Jukes-Cantor model (no CAT). To ensure that the simulated trees were resolvable, which facilitates comparison of methods (but inflates the accuracy of all methods), branch lengths of less than 0.001 were replaced with values of 0.001, which corresponds to roughly one substitution across the internal branch, as the 16S alignment has 1,287 positions. Evolutionary rates for each site were randomly selected from 16 rate categories according to a gamma distribution with a coefficient of variation of 0.7. Given the tree and the rates, sequences were simulated with Rose [34] under the HKY model and no transition bias. To allow Rose to handle branch lengths of less than 1%, we set “MeanSubstitution = 0.00134” and multiplied the branch lengths by 1,000.

### Software Used

We used FastTree 2.0.0. We used the July 6 2009 release of the PhyML 3.0 source code and modified BL_MIN from 1.e-10 to 1.e-8 to overcome numerical problems with some of the simulated protein alignments, as suggested by Stepháne Guindon. FastME 2.06 was provided by Olivier Gascuel. RAxML 7.0.4 and 7.2.1 were obtained from the author's web sites. RAxML 7.2.1 was compiled with SSE instructions. NINJA was provided by Travis Wheeler and is available at http://nimbletwist.com/software/ninja/. BIONJ was obtained from http://www.lirmm.fr/~w3ifa/MAAS/BIONJ/BIONJ.c. BIONJ was run with maximum-likelihood distances obtained with phylip's protdist (http://evolution.genetics.washington.edu/phylip.htm) and the JTT model (no gamma). Log-corrected distances were obtained with FastTree and the -makematrix option.

## Supporting Information

Figure S1 Branch lengths for an alignment of 200 16S rRNA sequences vary systematically with the Γ approximation used. The CAT lengths are from FastTree, and all Γ branch lengths are from PhyML with FastTree's topology and with optimized shape parameters. The top panel shows that branch lengths from the various models have a roughly linear relationship with each other, but they have different scales. The bottom panel shows how the total length of the tree varies with the number of categories (note log χ axis). The “Use Median” lengths are from running PhyML with –use_median, which uses the median of each region, rather than the mean, to approximate the gamma distribution. The “Corrected” lengths are the “Use Median” lengths multiplied by the average posterior rates, which can be obtained by running PhyML with –print_site_lnl (thanks to Stepháne Guindon for pointing this out). The corrected lengths converge to the correct value much more quickly than the other rates. The “CAT/Gamma” tree length, from FastTree 2.1 with -gamma, is also reasonably accurate. With this option, FastTree 2.1 optimizes the Γ_20_ likelihood with a shape parameter and a rescaling parameter, using the site likelihoods from FastTree's 20 relative rates and branch lengths that were optimized under the CAT model.
(0.13 MB PS)

Figure S2 Total Splits or Strongly Supported Splits that Disagree with RAxML's Final Tree, versus Time. The 16S tree has 4,111 splits and the COG trees have 2,497 splits each. All values for the COG trees are averages over the 7 COGs.
(0.02 MB PS)

Table S1 Times and likelihoods for large 16S rRNA alignments
(0.02 MB PDF)

## References

[1] Nawrocki EP, Kolbe DL, Eddy SR (2009). Infernal 1.0: inference of RNA alignments.. Bioinformatics.

[2] Price MN, Dehal PS, Arkin AP (2009). FastTree: computing large minimum evolution trees with profiles instead of a distance matrix.. Mol Biol Evol.

[3] Saitou N, Nei M (1987). The neighbor-joining method: a new method for reconstructing phylogenetic trees.. Mol Biol Evol.

[4] Studier JA, Keppler KJ (1988). A note on the neighbor-joining algorithm of Saitou and Nei.. Mol Biol Evol.

[5] Felsenstein J (1981). Evolutionary trees from dna sequences: A maximum likelihood approach.. J Mol Evol.

[6] Roch S (2006). A short proof that phylogenetic tree reconstruction by maximum likelihood is hard.. IEEE/ACM Trans Comput Biol Bioinform.

[7] Guindon S, Gascuel O (2003). A simple, fast, and accurate algorithm to estimate large phylogenies by maximum likelihood.. Syst Biol.

[8] Hordijk W, Gascuel O (2005). Improving the efficiency of SPR moves in phylogenetic tree search algorithms based on maximum-likelihood.. Bioinformatics.

[9] Stamatakis A (2006). RAxML-VI-HPC: maximum likelihood-based phylogenetic analyses with thousands of taxa and mixed models.. Bioinformatics.

[10] Felsenstein J (1985). Confidence limits on phylogenies: an approach using the bootstrap.. Evolution.

[11] Stamatakis A, Hoover P, Rougemont J (2008). A rapid bootstrap algorithm for the RAxML web servers.. Syst Biol.

[12] Desper R, Gascuel O (2002). Fast and accurate phylogeny reconstruction algorithms based on the minimum-evolution principle.. Journal of Computational Biology.

[13] Nei M, Kumar S, Takahashi K (1998). The optimization principle in phylogenetic analysis tends to give incorrect topologies when the number of nucleotides or amino acids used is small.. Proc Natl Acad Sci USA.

[14] Stamatakis A (2006). Phylogenetic models of rate heterogeneity: a high performance computing perspective..

[15] Yang Z (1994). Maximum likelihood phylogenetic estimation from DNA sequences with variable rates over sites: Approximate methods.. J Mol Evol.

[16] Guindon S, Delsuc F, Dufayard JF, Gascuel O (2009). Estimating maximum likelihood phylogenies with PhyML.. Methods Mol Biol.

[17] Guindon S, Dufayard J, Lefort V, MAnisimova, Hordijk W (2010). New algorithms and methods to estimate maximum-likelihood phylogenies: Assessing the performance of PhyML 3.0.. Syst Biol.

[18] Gascuel O (1997). BIONJ: an improved version of the NJ algorithm based on a simple model of sequence data.. Mol Biol Evol.

[19] Shimodaira H, Hasegawa M (1999). Multiple comparisons of log-likelihoods with applications to phylogenetic inference.. Mol Biol Evol.

[20] Tatusov RL, Natale DA, Garkavtsev IV, Tatusova TA, Shankavaram UT (2001). The COG database: new developments in phylogenetic classification of proteins from complete genomes.. Nucleic Acids Res.

[21] Anisimova M, Gascuel O (2006). Approximate likelihood-ratio test for branches: A fast, accurate, and powerful alternative.. Syst Biol.

[22] Galtier N, Jean-Marie A (2004). Markov-modulated markov chains and the covarion process of molecular evolution.. J Comput Biol.

[23] DeLong ER, Clarke-Pearson DL (1998). Comparing the areas under two or more correlated receiver operating characteristic curves: a nonparametric approach.. Biometrics.

[24] Evans J, Sheneman L, Foster J (2006). Relaxed neighbor joining: a fast distance-based phylogenetic tree construction method.. J Mol Evol.

[25] Wheeler TJ (2009). Large-scale neighbor-joining with NINJA..

[26] Desper R, Gascuel O (2004). Theoretical foundation of the balanced minimum evolution method of phylogenetic inference and its relationship to weighted least-squares tree fitting.. Mol Biol Evol.

[27] Bordewich M, Gascuel O, Huber KT, Moulton V (2009). Consistency of topological moves based on the balanced minimum evolution principle of phylogenetic inference.. IEEE/ACM Trans Comput Biol Bioinform.

[28] Shimodaira H, Hasegawa M (2001). CONSEL: for assessing the confidence of phylogenetic tree selection.. Bioinformatics.

[29] Goloboff PA, Farris JS, Nixon KC (2008). TNT, a free program for phylogenetic analysis.. Cladistics.

[30] Katoh K, Toh H (2007). PartTree: an algorithm to build an approximate tree from a large number of unaligned sequences.. Bioinformatics.

[31] Bradley RK, Roberts A, Smoot M, Juvekar S, Do ea J (2009). Fast statistical alignment.. PLoS Comput Biol.

[32] Sonnhammer ELL, Hollich V (2005). Scoredist: A simple and robust protein sequence distance estimator.. BMC Bioinformatics.

[33] DeSantis TZ, Hugenholtz P, Larsen N, Rojas M, Brodie EL (2006). Greengenes, a chimera-checked 16S rRNA gene database and workbench compatible with ARB.. Appl Environ Microbiol.

[34] Stoye J, Evers D, Meyer F (1998). Rose: generating sequence families.. Bioinformatics.

