# Supplementary material for: FastTree 2 – Approximately Maximum-Likelihood Trees for Large Alignments
Source: PLoS One. 2010 Mar 10;5(3):e9490. doi: 10.1371/journal.pone.0009490 (PMC2835736; doi:10.1371/journal.pone.0009490)
Supplement: Table S1 — Times and likelihoods for large 16S rRNA alignments (0.02 MB PDF) [file pone.0009490.s003.pdf]

**#Sequences**	2,000		4,114		6,718		15,011	
**Method**	**Sec**	**Log-Lk**	**Sec**	**Log Lk**	**Sec**	**Log Lk**	**Sec**	**Log Lk**
FastTree, no ML	84	-368,567	210	-330,774	348	-499,732	1,082	-3,981,266
FastTree with ML	265	-366,517	585	-328,062	955	-493,841	2,441	-3,961,198
no ML + 1 round SPRs	576	-366,128	1,154	-327,193	2,085	-492,522	4,499	-3,955,028
no ML + 2 rounds SPRs	973	-365,766	2,003	-326,786	3,253	-489,991	6,245	-3,948,824
RAxML Parsimony	286	-366,605	740	-328,069	1,264	-484,909	7,764	-3,951,842
+1 round SPRs	943	-365,745	1,832	-326,854	3,559	-482,927	12,467	-3,940,585
+2 rounds SPRs	1,307	-365,617	2,576	-326,584	4,685	-482,530	14,126	-3,937,613
RAxML, Final	12,618	-364,912	23,637	-325,581	44,905	-481,259	106,855	-3,924,702

This is the same analysis as in Figure 2, but in tabular form, and with data for additional 16S rRNA alignments.
